# Mouse double minute 2 (MDM2) upregulates Snail expression and induces epithelial-to-mesenchymal transition in breast cancer cells *in vitro* and *in vivo*

**DOI:** 10.18632/oncotarget.9287

**Published:** 2016-05-11

**Authors:** Xiangdong Lu, Caiyun Yan, Yi Huang, Dongmin Shi, Ziyi Fu, Jinrong Qiu, Yongmei Yin

**Affiliations:** ^1^ Department of Oncology, The First Affiliated Hospital of Nanjing Medical University, Nanjing, P. R. China; ^2^ Department of Pharmacology and Chemical Biology, Magee Women's Research Institute, University of Pittsburgh Cancer Institute, Pittsburgh, PA, USA; ^3^ Nanjing Maternal and Child Medical Institute, Affiliated Nanjing Maternity and Child Health Care Hospital, Nanjing Medical University, Nanjing, P. R. China

**Keywords:** breast cancer, MDM2, epithelial-mesenchymal transition, Snail

## Abstract

The oncogene, mouse double minute 2 (*MDM2*), has been implicated in the pathogenesis of numerous cancers. In this study, we investigated the role of MDM2 in epithelial-to-mesenchymal transition (EMT) and the underlying mechanisms in breast cancer cells *in vitro* and *in vivo*. The results showed that up-regulation of *MDM2* in MCF-7 cells altered the cell morphology to a mesenchymal phenotype. Knockdown of *MDM2* in MDA-MB-231 cells altered the cell morphology to the epithelial phenotype. In addition, overexpression of *MDM2* increased the expression of N-cadherin and Vimentin and decreased the expression of E-cadherin, at both the mRNA and protein levels, *in vitro* and *in vivo*. Conversely, down-regulation of *MDM2* decreased the expression of N-cadherin and Vimentin, and increased the expression of E-cadherin *in vitro*. Furthermore, MDM2 up-regulated both the mRNA and protein expression of Snail *in vitro* and *in vivo*. Knockdown of Snail almost abolished MDM2 induced EMT *in vitro*. Finally, we found that MDM2 expression correlated with EMT markers and Snail: Snail expression was inversely associated with E-cadherin in human breast cancer samples. Our findings demonstrated that MDM2 induces EMT by enhancing Snail expression *in vitro* and *in vivo*. Thus, MDM2 may be a potential target for therapy against human metastatic breast cancer.

## INTRODUCTION

Globally, breast cancer is the most common malignancy and the second leading cause of cancer deaths in female [1–3]. During recent decades, world cancer reports have shown a continuously high breast cancer incidence in western countries and a gradually increasing trend in developing countries. Metastasis is the main cause of fatality in breast cancer patients [4]. It has been estimated that 25%–40% of patients with breast cancer will develop metastatic disease, which is generally incurable [5]. The 5-year survival rate for metastatic breast cancer patients is 20% [6]. Therefore, unraveling the molecular mechanisms underlying breast cancer metastasis is imperative.

The mouse double minute 2 (MDM2) protein is a multifunctional oncoprotein that has both p53-dependent and independent roles in oncogenesis. The *MDM2* gene amplification occurs in diverse human malignancies, including soft tissue sarcomas and cancers of the brain, breast, ovary, cervix, lung, colon, prostate and kidney [7–9]. Moreover, studies have shown that *MDM2* overexpression is associated with tumors that have a higher degree of invasiveness, later stages, greater metastatic potential and resistance to chemotherapeutic agents and radiation [10]. In our previous study [11], we demonstrated that MDM2 promotes invasion and metastasis of breast cancer by upregulating *MMP9* expression, causing increased extracellular matrix breakdown. Whether MDM2 influences other process of breast cancer metastasis requires further exploration.

A well-recognized mechanism for initiating tumor cell invasive and metastatic behavior is epithelial-mesenchymal transition (EMT), in which polarized epithelial breast cancer cells acquire a motile mesenchymal phenotype [12, 13]. Important hallmarks of EMT include the decreased expression of the epithelial marker E-cadherin, and increased expression of mesenchymal markers, such as N-cadherin and Vimentin [14]. Snail, a zinc-finger transcription factor, has a pivotal role in EMT as a repressor of E-cadherin [15]. EMT assists the tumor cells to invade the local matrix and enter into blood vessels, which finally form distant metastasis in other sites [16]. Thus, considering EMT's role at the onset of the metastatic process, controlling EMT in tumors is considered a promising strategy to inhibit metastasis and improve survival of cancer patients.

The purpose of this study was to explore the role and the underlying mechanisms of MDM2 in EMT. We found that overexpression of *MDM2* caused the occurrence of EMT and knockdown of *MDM2* led to mesenchymal-epithelial transition (MET) in breast cancer cells *in vitro* and *in vivo*. Moreover, we found that promotion of EMT by MDM2 functioned by upregulating Snail. Our study proved that MDM2 has a critical role in inducing EMT, thus promoting breast cancer metastasis.

## RESULTS

### MDM2 was highly expressed in invasive human breast cancer cell lines

**Figure 1:**
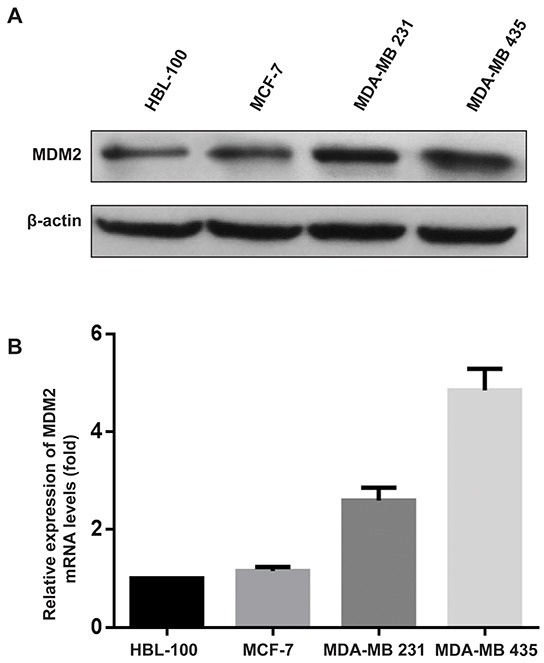
*MDM2* was highly expressed in invasive human breast cancer cell lines *MDM2* expression was examined by western blot analysis **A.** and qRT-PCR **B.** in human breast cancer cell lines. For the western blot analysis, β-actin was used as the loading control. For qRT-PCR, *GAPDH* served as an internal control.

To develop *in vitro* models to examine the mechanism of MDM2's function in breast cancer biology, we determined the protein expression of MDM2 in three human breast cancer cell lines (MCF-7, MDA-MB-231 and MDA-MB-435) and one human mammary epithelial cell (HBL-100) by western blotting. The results showed that MDM2 was highly expressed in two invasive breast cancer cells (MDA-MB-231 and MDA-MB-435) compared with the noninvasive breast cancer cell (MCF-7) and mammary epithelial cell (HBL-100). Quantitative real-time reverse transcription PCR (qRT-PCR) analysis confirmed that *MDM2* mRNA expression correlated with the protein expression in these cell lines. Similar to the data shown in Figure 1A, MDA-MB-435 showed the highest *MDM2* mRNA expression and MCF-7 was among the breast cancer cells with the lowest *MDM2* mRNA expression (Figure 1B).

### Generation of stable cell lines

**Figure 2:**
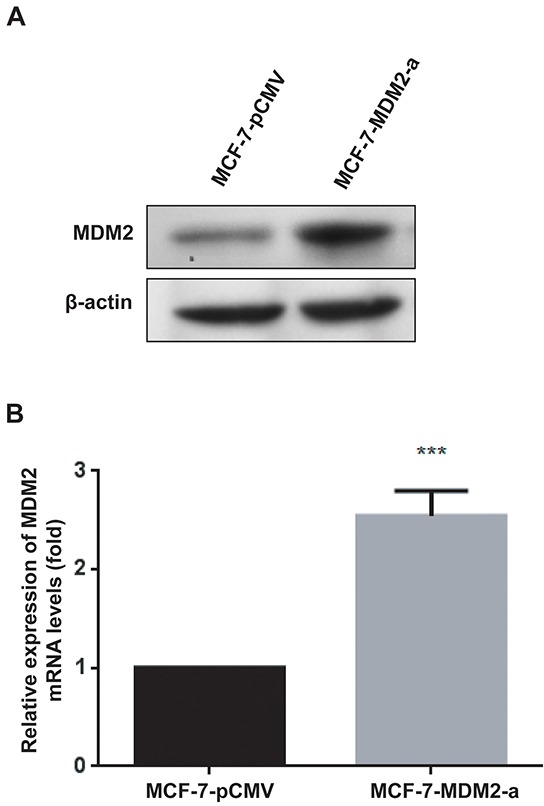
Generation of stable cell lines MDM2 protein expression was evaluated by western blotting in MCF-7-pCMV and MCF-7-MDM2-a cells **A.** β-actin was used as the loading control. *MDM2* mRNA expression was analyzed by qRT-PCR in MCF-7-pCMV and MCF-7-MDM2-a cells **B.** *GAPDH* served as the internal control. ***P<0.001. The results are from three independent experiments. Error bars indicate the standard deviation.

To determine the effects of MDM2 on the biological behavior of breast cancer cells, MCF-7 cells were infected with pRDI292-CMV or pRDI292-CMV-MDM2 lentiviruses, and the sub-clonal cells were established by puromycin selection. The stable overexpression of MDM2 in MCF-7 cells (designated as MCF-7-MDM2-a and MCF-7-MDM2-d) and the control (designated as MCF-7-pCMV) were established. The levels of *MDM2* mRNA and protein expression in these resultant cell lines were examined by qRT-PCR and western blotting. As shown in Figure 2A and Supplementary Figure S1A, MDM2 could be detected in MCF-7-pCMV cells, whereas MDM2 expression was significantly increased in MCF-7-MDM2-a and MCF-7-MDM2-d cells. The expression of *MDM2* mRNA is shown in Figure 2B and Supplementary Figure S1B. These results indicated that the recombinant lentivirus used in this study was efficient to express MDM2 in the MCF-7 cells.

### MDM2 overexpression promotes EMT in MCF-7 cells

**Figure 3:**
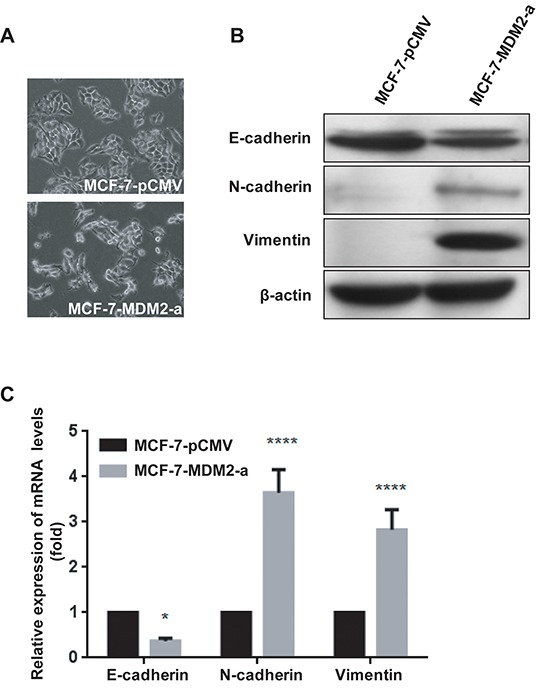
MDM2 overexpression promotes EMT in MCF-7 cells Representative phase-contrast images of MCF-7-pCMV and MCF-7-MDM2-a cells showed MDM2-associated morphological changes **A.** (200×). Expression of epithelial and mesenchymal markers was evaluated by western blotting in MCF-7-pCMV and MCF-7-MDM2-a cells **B.** β-actin was used as the loading control. Expression of epithelial and mesenchymal markers was analyzed by qRT-PCR in MCF-7-pCMV and MCF-7-MDM2-a cells **C.** *GAPDH* served as an internal control. *P<0.05 and ****P<0.0001. The results are from three independent experiments. Error bars indicate the standard deviation.

To investigate whether the overexpression of MDM2 altered the functions of the MCF-7 cells, we observed the morphological changes and found that MCF-7-pCMV cells exhibited a cobblestone-like appearance, whereas MCF-7-MDM2-a cells displayed a scattered and more mesenchymal-like morphology (Figure 3A). We then examined the levels of EMT markers, such as E-cadherin, N-cadherin and Vimentin in both the MCF-7-MDM2-a cells and MCF-7-pCMV cells. As shown in Figure 3B, the expression of the epithelial marker (E-cadherin) decreased, whereas the levels of the mesenchymal markers (N-cadherin and Vimentin) increased in MCF-7-MDM2-a cells. Simultaneously, the expression levels of the mRNAs correlated with the corresponding protein levels (Figure 3C), indicating that MDM2 decreased and increased the expression of epithelial and mesenchymal markers, respectively, at the transcript level. Similar results were observed in MCF-7-MDM2-d cells (Supplementary Figure S2).

### Knockdown of *MDM2* promotes MET in MDA-MB-231 cells

**Figure 4:**
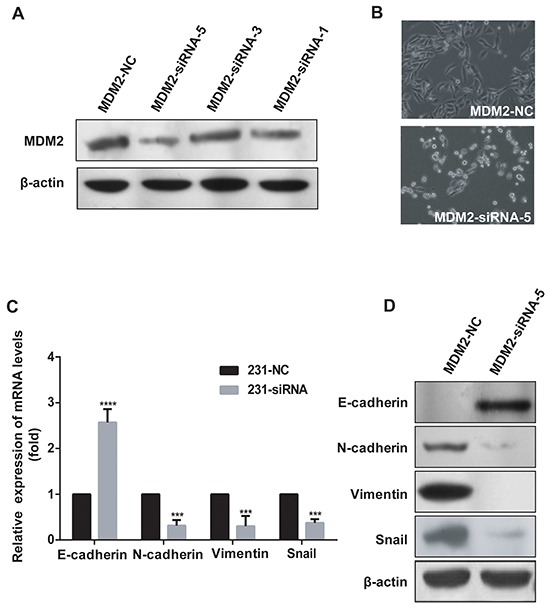
*MDM2* knockdown promotes MET in MDA-MB-231 cells MDM2-siRNA-5 had the highest inhibition efficiency and MDM2-siRNA-1 had the second highest inhibition efficiency **A.** Knockdown of *MDM2* in MDA-MB-231 cells induced morphological changes to an epithelial phenotype (200×) **B.** In 231-MDM2-siRNA-5 cells, qRT-PCR analyses showed increased E-cadherin and decreased N-cadherin, Vimentin and Snail mRNA levels, compared with the control cells (231-MDM2-NC) **C.** In 231-MDM2-siRNA-5 cells, western blotting showed decreased N-cadherin, Vimentin, Snail and increased E-cadherin protein levels **D.** ***P<0.001 and ****P<0.0001. The results are from three independent experiments. Error bars indicate the standard deviation.

To determine the role of MDM2 in EMT further, we knocked down *MDM2* using siRNAs in MDA-MB-231 cells, which expresses endogenous N-cadherin and Vimentin. As shown in Figure 4A, MDM2-siRNA-5 and MDM2-siRNA-1 had higher inhibition efficiencies than the other siRNAs. We then observed that *MDM2* knockdown induced MDA-MB-231 cells to acquire an epithelial phenotype (Figure 4B). Furthermore, we observed a dramatic decrease in N-cadherin, Vimentin and Snail mRNA levels by qRT-PCR analysis when *MDM2* was knocked down (P<0.05). In contrast, E-cadherin showed a significant increase in expression (P<0.05) (Figure 4C). Strikingly, the E-cadherin, N-cadherin, Vimentin and Snail protein expression levels showed the same trend as their mRNAs (P<0.05). This situation was more physiologically relevant than the situation of overexpression. Notably, we did not detect any E-cadherin expression in MDA-MB-231 cells, but we detected an upregulation of E-cadherin in 231-MDM2-siRNA-5 cells (Figure 4D). Taken together, these results indicated that knockdown of *MDM2* led to MET in MDA-MB-231 cells. Similar results were observed in MDA-MB-231 cells transfected with MDM2-siRNA-1 (Supplementary Figure S3).

### MDM2 induces EMT by upregulating the expression of Snail

**Figure 5:**
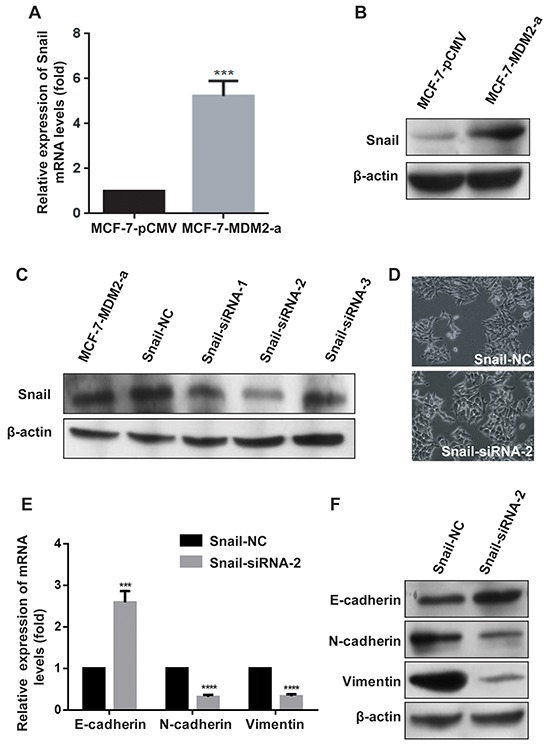
MDM2 induces EMT by upregulating the expression of Snail Overexpression of *MDM2* increased the expression of Snail in MCF-7 cells at the mRNA levels **A.** Overexpression of MDM2 increased the expression of Snail in MCF-7 cells at the protein levels **B.** Snail-siRNA-2 had the highest inhibition efficiency **C.** Knockdown of Snail in MCF-7-MDM2-a cells induced morphological changes to an epithelial phenotype (200×) **D.** In MCF-7-MDM2-a cells, qRT-PCR analyses showed that knockdown of Snail increased the expression of E-cadherin and decreased the expression of N-cadherin and Vimentin at mRNA levels **E.** In MCF-7-MDM2-a cells, western blotting showed that knockdown of Snail increased the expression of E-cadherin and decreased the expression of N-cadherin and Vimentin proteins **F.** Knocking down Snail transiently and then, after 24 h, transfecting pCMV-MDM2 expression plasmids in MCF-7 cells induced protein expression changes (evaluated by western blotting). **G.** and mRNA expression changes (analyzed by qRT-PCR), **H.** NF-κB p65-siRNA-3 had the highest inhibition efficiency **I.** Knocking down NF-κB p65 subunit transiently and then, after 24 h, transfecting pCMV-MDM2 expression plasmids in MCF-7 cells induced protein expression changes (evaluated by western blotting), **J.** and mRNA expression changes (analyzed by qRT-PCR), **K.** β-actin was used as the loading control. GAPDH served as an internal control (qRT-PCR). **P<0.01, ***P<0.001, **** P<0.0001. The results are from three independent experiments. Error bars indicate the standard deviation.

Snail, a zinc finger-type transcription factor, is the most prominent EMT transcriptional regulator and contributes to EMT mainly by acting as an E-cadherin repressor [17, 18]. We examined whether Snail was activated by MDM2. The results shown in Figure 5A and 5B revealed that both the mRNA and protein levels of Snail were elevated in MCF-7-MDM2-a cells compared with MCF-7-pCMV cells. To verify the role of Snail in the EMT process triggered by MDM2, Snail was knocked down by RNA interfering technology. Figure 5C shows that Snail-siRNA-2 had the highest inhibition efficiency. Figure 5D shows that in MCF-7-MDM2-a cells, MDM2-induced morphological changes were almost abolished when Snail was knocked down. In addition, the expressions of E-cadherin/N-cadherin/Vimentin were almost reversed compared to the Snail-NC groups (Figure 5E, 5F). Moreover, we found that the EMT-promoting ability of MDM2 in MCF-7 cells was attenuated by knockdown of Snail (Figure 5G, 5H). These results indicated that Snail mediated the MDM2-induced EMT by acting as an E-cadherin repressor in MCF-7-MDM2-a cells. Furthermore, we observed that the upregulation of Snail induced by MDM2 was impaired by knockdown of NF-kappaB/p65 (Figure 5I–5K), indicating that NF-kappaB/p65 was involved in the MDM2-induced upregulation of Snail.

### MDM2 promotes tumor growth, induces EMT and confers metastatic potential on human breast cancer cells

**Figure 6:**
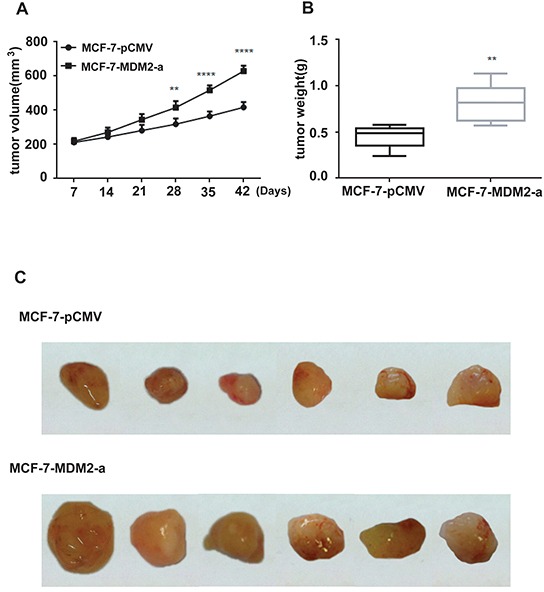
Effect of MDM2 on tumor growth of experimental breast cancer MCF-7-pCMV or MCF-7-MDM2-a cells were subcutaneously implanted into nude mice. All mice underwent monitoring of tumor growth. The tumor size was measured twice weekly for 6 weeks. The mice were sacrificed on day 42, and the tumors were resected and weighted. Xenograft tumor size **A.** Xenograft tumor weight **B.** Representative pictures from each group **C.** Data represent mean ± SD; n=6 mice/group. **p<0.01, ****p<0.0001.

**Figure 7:**
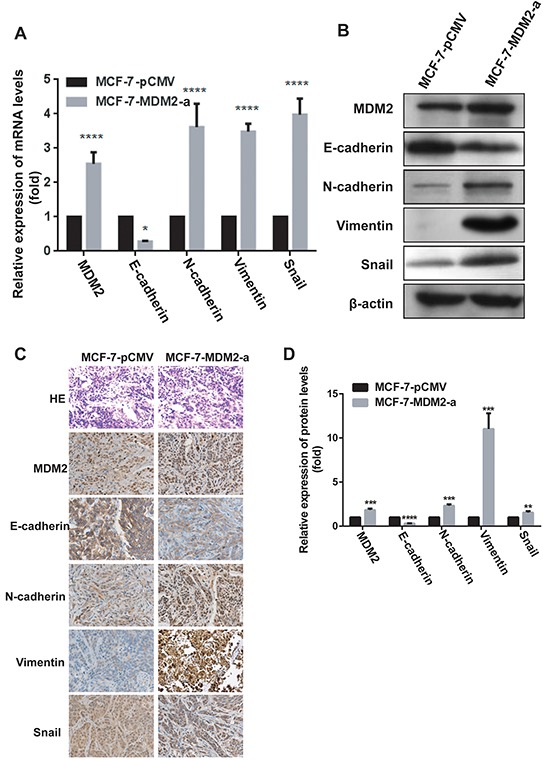
MDM2 induces EMT and confers metastatic potential of human breast cancer through the up-regulation of Snail *in vivo* The expression of *MDM2*, EMT markers and Snail mRNA levels were analyzed by qRT-PCR *in vivo* **A.** The expression of MDM2, EMT markers and Snail protein levels were analyzed by western blotting **B.** and immunohistochemical analysis **C, D.** (400×) *in vivo*. *p<0.05, **P<0.01, ***P<0.001, and ****P<0.0001. Error bars indicate the standard deviation.

To evaluate the effects of MDM2 overexpression on EMT *in vivo*, we xenografted the MCF-7-MDM2-a and MCF-7-pCMV cells into nude mice. As shown in Figure 6A, 6B, 6C, tumors derived from MCF-7-MDM2-a cells grew much faster and weighed significantly more than those formed from MCF-7-pCMV cells. We then detected the levels of *MDM2*, Snail, E-cadherin, N-cadherin and vimentin mRNA and protein in tumors derived from nude mice by qRT-PCR, western blotting and immunohistochemical analysis. In agreement with our *in vitro* data, at the mRNA level, *MDM2* expression significantly increased in tumors formed from MCF-7-MDM2-a cells. Meanwhile, tumors formed from MCF-7-MDM2-a cells showed increased expression of N-cadherin and vimentin, and decreased expression of E-cadherin. Furthermore, tumors formed from MCF-7-MDM2-a cells showed increased expression of Snail (Figure 7A). Western blotting and immunohistochemical analysis also indicated that tumors formed from MCF-7-MDM2-a groups had increased levels of MDM2, N-cadherin, Vimentin and Snail proteins, but decreased levels of E-cadherin protein (Figure 7B, 7C, 7D).

### MDM2 expression correlated with EMT markers and Snail in human breast cancer

**Figure 8:**
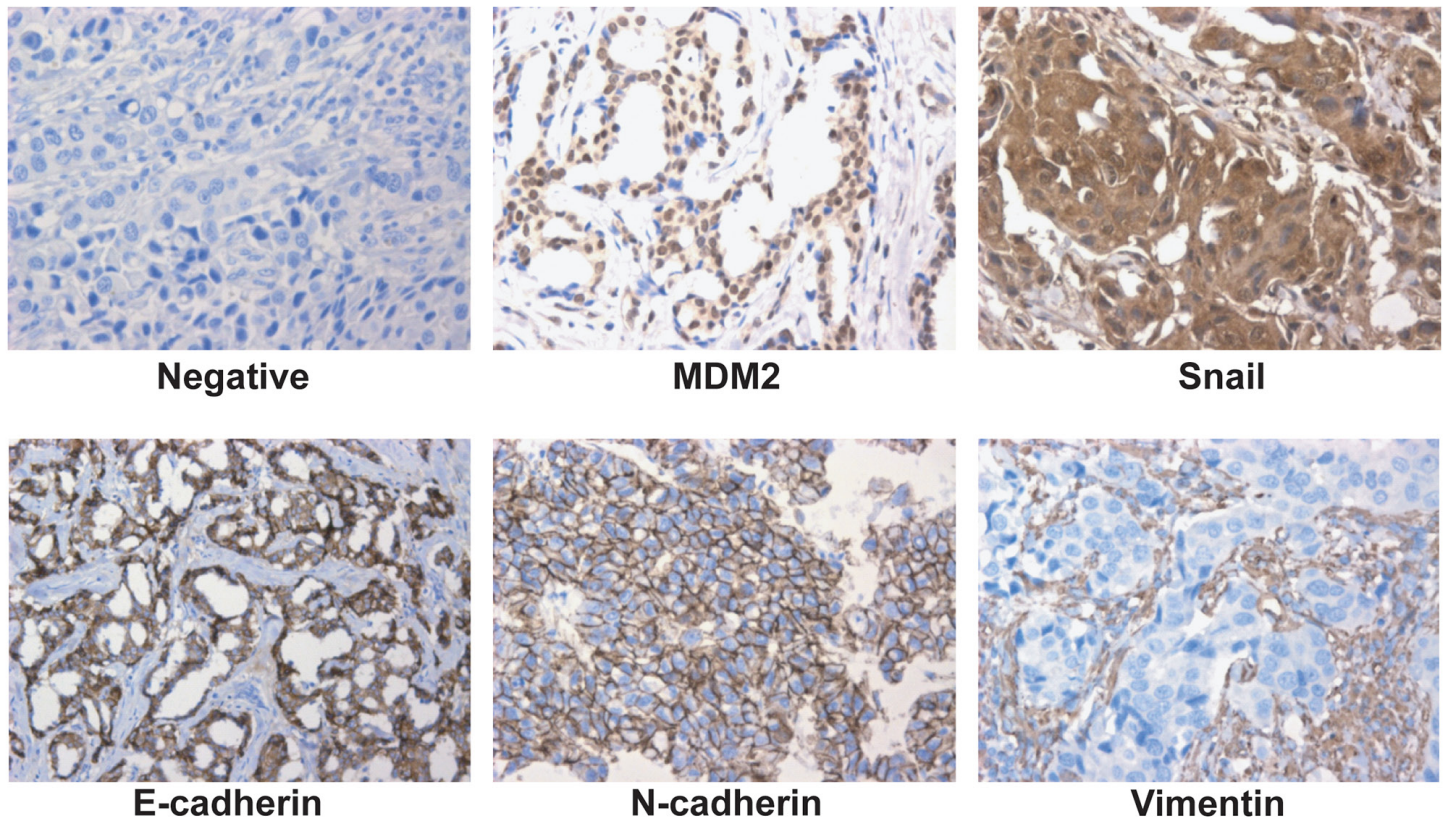
Expression of MDM2, Snail and EMT markers in human breast cancer Representative fields of view from the TMA cores show examples of negative staining patterns, positive MDM2, Snail, E-cadherin, N-cadherin and Vimentin (400×). MDM2 exhibited nuclear or cytoplasmic immunoreactivity, Snail exhibited nuclear immunoreactivity; E-cadherin, N-cadherin and Vimentin exhibited membrane or cytoplasmic immunoreactivity.

In the analysis of a 62-member tissue microarray (TMA), MDM2 expression was detected in 30 cases (48.4%); 32 tumors (51.6%) did not express MDM2. To explore the correlation, we then stained for the expression of four genes (E-cadherin, N-cadherin, Vimentin and Snail) that are associated with EMT (Figure 8). Spearman correlation analysis confirmed that the immunohistochemical expression of MDM2 inversely correlated with E-cadherin. MDM2 expression directly correlated with N-cadherin and Vimentin (Table 1). Furthermore, MDM2 expression directly correlated with Snail, but Snail expression was inversely correlated with E-cadherin (Table 2). Therefore, MDM2 may promote EMT of breast cancer via upregulating Snail.

**Table 1:** Correlation among the expressions of MDM2 and EMT markers (E-cadherin, N-cadherin, Vimentin) in human breast cancer [n (%)]

EMT markers	n	MDM2 expression		Spearman correlation	
		negative	positive	value(r)	P value
E-cadherin					
negative	18	2 (11.1)	16 (88.9)	−0.518	0.000
positive	44	30 (68.2)	14 (31.8)		
N-cadherin					
negative	57	32 (56.1)	25 (43.9)	0.306	0.016
positive	5	0 (0.0)	5 (100.0)		
Vimentin					
negative	42	27 (64.3)	15 (35.7)	0.367	0.003
positive	20	5 (25.0)	15 (75.0)		

The spearman correlation was used to compare the degrees of correlation. Positive numbers reflect direct correlation, and negative numbers reflect inverse correlation.

**Table 2:** Correlation among Snail, MDM2 and E-cadherin expression markers in human breast cancer [n (%)]

	n	Snail expression		Spearman correlation	
		negative	positive	value(r)	P value
MDM2					
negative	32	23 (71.9)	9 (28.1)	0.354	0.005
positive	30	11 (36.7)	19 (63.3)		
E-cadherin					
negative	18	2 (11.1)	16 (88.9)	−0.562	0.000
positive	44	32 (72.7)	12 (27.3)		

The spearman correlation was used to compare the degrees of correlation. Positive numbers reflect direct correlation, and negative numbers reflect inverse correlation.

## DISCUSSION

The *MDM2* gene, located on chromosome 12q13-14 with a genomic size of 34 kb, was first cloned as an amplified gene on a murine double-minute chromosome in the 3T3DM cell line, a spontaneously transformed derivative of BALB/c 3T3 cells [19]. Structurally, the MDM2 protein contains several highly conserved functional domains, including an N-terminal domain, a central acidic domain adjacent to a zinc finger domain, and a C-terminal RING finger domain. MDM2 is structurally or functionally related to certain critical molecules, such as Rb, E2F, p21, p19^arf^ and ras, thus connecting the main signal pathways controlling the balance between proliferation and apoptosis [20, 21]. In a previous study, we provided insight into the mechanisms and functions of MDM2 in invasion [11].

In the present study, we focused on EMT, because understanding its molecular mechanisms is crucial to developing new therapeutic strategies against breast cancer invasiveness and metastatic dissemination of carcinoma cells. EMT is believed to be a key step during cancer metastasis. In the progression of malignancy, EMT also enables carcinoma cells to lose their epithelial adherence, undergo cytoskeleton remodeling, facilitate their detaching from the tumor mass, migrate to distant sites, and eventually form metastatic tumor masses. *MDM2* overexpression is associated with a more aggressive phenotype and decreased overall survival, and contributes to migration and invasion in breast cancer [11]. Therefore, we hypothesized that MDM2 and EMT are associated in breast cancer. Snail transcription factor is a C2H2 zinc finger protein that promotes EMT, which involves a loss of epithelial markers, such as E-cadherin, and an increase in mesenchymal markers, such as vimentin [22–25]. Snail transcriptionally represses genes by binding to the enhancer sequence (E-box) of genes such as E-cadherin, Occludin, Claudins, and Mucin-1 [22, 23]. Moreover, Snail induces resistance to cell death, which was noted in skin tumors induced in mice, biopsies of breast carcinomas from patients, gastric cancer, and hepatocellular carcinomas [26].

In this research, studies in cell line models showed that the overexpression of *MDM2* induced EMT in MCF-7 cells *in vitro* and *in vivo*. By contrast, *MDM2* knockdown induced MET in MDA-MB-231 cells *in vitro*. Further mechanistic studies in MCF-7 cells identified Snail as a target of MDM2 and showed that Snail mediates MDM2-induced EMT by downregulating E-cadherin. Thus, a mechanism involving MDM2 control of EMT in MCF-7 cells was identified. Our findings demonstrated a central function for MDM2 in the regulatory mechanism of EMT in breast cancer and expanded the repertoire of pathways that participate in this cellular process.

In conclusion, our results showed that MDM2 overexpression induces EMT, which may contribute to breast cancer metastasis. Furthermore, MDM2 induces EMT via upregulation of Snail. These data implied that MDM2 has important roles in the mechanism of breast cancer progression and may serve as a therapeutic target for breast cancer.

## MATERIALS AND METHODS

### Chemicals and reagents

Human breast cancer cells MCF-7 and MDA-MB-231 were from the Type Culture Collection of the Chinese Academy of Sciences (Shanghai, China). The Dulbecco's modified eagle medium (DMEM) medium and fetal bovine serum (FBS) were purchased from Gibco (BRL Grand Island, NY, USA). Antibodies for E-cadherin and Vimentin were obtained from Cell Signaling Technology (Beverly, MA, USA). Antibodies for N-cadherin and MDM2 were purchased from Santa Cruz Biotechnology (Santa Cruz, CA, USA). Antibodies for Snail and NF-kappaB/p65 were obtained from Abcam (Cambridge, UK). SYBR Premix Ex Taq™ was from Takara (Shiga, Japan). Horseradish peroxidase (HRP)-conjugated secondary antibody, Trizol reagent and Lipofectamine 2000 were purchased from Invitrogen (Carlsbad, CA, USA). The BCA Protein Assay Kit was from Thermo (Waltham, USA). Small interfering RNAs (siRNA) against human MDM2 (siMDM2, Accession number: NM_002392) and human Snail (siSnail, Accession number: NM_005985) and scrambled controls (siNC) were purchased from Genechem (Shanghai, China). Small interfering RNAs (siRNA) against human NF-kappaB/p65 (siNF-kappaB/p65, Accession number: NM_021975) and scrambled control (siNC) were from Ribobio (Guangzhou, China). 17β-Estradiol pellets were obtained from Innovative Research (Sarasota, FL, USA). Matrigel was purchased from BD Biosciences (Bedford, MA, USA). Lentiviruses encoding MDM2 and the control were constructed and examined by Shengbo (Shanghai, China). The nude mice were obtained from the Model Animal Research Center of Nanjing University (Nanjing, China).

### Construction of *MDM2* overexpression stable cell lines

Briefly, MCF-7 cells were exposed to lentivirus-containing supernatant for 24 h in the presence of polybrene. Puromycin (2mg/ml) selection was performed to select cells with stable pRDI292-CMV (delivering control or human MDM2 cDNA) genomic integration. Fluorescence-activated cell sorting was used to select cells with stable pSicoR constructs, based on GFP expression. Finally, stable expression of *MDM2* was verified by qRT-PCR and western blotting. MDM2 overexpression stable cell lines were named MCF-7-MDM2-a, MCF-7-MDM2-d and the control was named MCF-7-pCMV.

### Cell culture

HBL-100, MCF-7, MDA-MB-231, MDA-MB-435, MCF-7-MDM2 and MCF-7-pCMV cells were cultured in DMEM medium supplemented with 10% FBS, 100 U/ml penicillin, and 100 μg/ml streptomycin in a humidified 5% CO_2_ and 95% air atmosphere at 37°C.

### RNA extraction, reverse transcription and qRT-PCR

Total RNA was extracted from cultured cells using the Trizol reagent, according to the manufacturer's protocol. cDNA was synthesized using SuperScript II Reverse Transcriptase. Expression of genes was detected by qRT-PCR using SYBR Premix ExTaq. Normalization involved the 2^−ΔΔCT^ method relative to *GAPDH* expression. Primer sequences for MDM2 were: forward, 5′-CACGCCACTTTTTCTCTGCT-3′ and reverse, 5′-CCT GATCCAACCAATCACCT-3′; E-cadherin, forward, 5′-GA CCG-AGAGAGTTTCCCTACG-3′ and reverse, 5′-TCAG GCACCTGACCCTTGTA-3′; N-cadherin, forward, 5′-GA GATCCTACTGGACGGTTCG-3′ and reverse, 5′-TCTTG GCGAATGATCTTAGGA-3′; Vimentin, forward, 5′-CCTT GAACGCAA-AGTGGAATC-3′ and reverse, 5′-TGAGG TCAGGCTTGGAAACAT-3′; Snail, forward, 5′-CTGCG GGAAGGCCTTCTCT-3′ and reverse, 5′-CGCCTGGCA CTGG-TACTTCTT-3′; GAPDH, forward, 5′-GGTCTCC TCTGACTTCAACA-3′ and reverse, 5′-AGCCAAATT CGTTGTCATAC-3′.

### Western blot analysis

Total cells were homogenized in RIPA buffer [50 mM Tris-HCl(pH 7.5), 150 mM NaCl, 1% NP-40, 0.5% sodium deoxycholate and 0.1% SDS] containing protease inhibitors and centrifuged at 14,000×g for 30 min. The supernatant was recovered and proteins were quantified using the BCA Protein Assay Kit. Equal amounts of protein extracts were separated by 8% SDS-polyacrylamide gel electrophoresis (PAGE) and subsequently transferred onto polyvinylidene difluoride (PVDF) membranes. After blocking in fresh blocking buffer [0.1% Tween20 in Tris-buffered saline (TBS-T) containing 5% fat-free milk] at room temperature (RT) for 1.5 h, the membranes were incubated with the primary antibody overnight at 4°C. The membranes were then washed three 10 min in TBS-T and incubated with HRP-conjugated individual secondary antibody for 1.5 h at room temperature, followed by three 10 min washes in TBS-T. Finally, the signals were developed using the ECL reagent. Prestained markers were used as molecular weight standards. Each experiment was repeated at least three times.

### Gene knockdown

The siRNA of MDM2 (siMDM2), siRNA of Snail (siSnail), siRNA of NF-kappaB/p65 (sip65) and random non-coding RNA (siNC) were transfected into MDA-MB-231, MCF-7 or MCF-7-MDM2 cells using Lipofectamine 2000, according to the manufacturer's instruction (Invitrogen). The efficiency of genetic silencing by the siRNAs was evaluated by qRT-PCR (after 36 h transfection) and western blotting (after 48 h transfection).

### Breast cancer tissue microarrays

Tissue microarrays including 62 normal representative paraffin embedded tissue samples from 62 patients with breast cancer were created using a previously reported protocol [27] by the Shanghai Biochip Center. The paraffin embedded tissue samples were obtained from the First Affiliated Hospital of Nanjing Medical University in Nanjing. Written informed consent was obtained from each patient, and the study was approved by the Institute Research Ethics Committee at the First Affiliated Hospital of Nanjing Medical University. No patient received any other therapy before surgery. The tumor stage was determined according to the 2010 American Joint Committee on Cancer and International Union against cancer tumor-node-metastasis (TNM) classification system. All tumor samples were acquired at the time of operation and fixed with paraformaldehyde (4%).

### Immunohistochemical analysis

After deparaffinization and rehydration of the sections from paraffin-embedded tissue specimens, antigen retrieval was performed in a pressure cooker. Endogenous peroxidase activity was blocked with 0.5% hydrogen peroxide for 10 min. Antigens were recovered by heating the slides in an autoclave sterilizer for 2 min in 0.01 mol/l Tris-HCl at pH 6.0. The sections were incubated overnight at 4°C with a primary anti-MDM2 antibody (1:100), anti-E-cadherin antibody (1:200), anti-N-cadherin antibody (1:100), anti-Vimentin antibody (1:200) or anti-Snail antibody (1:200). The sections were incubated with the secondary antibody for 30 min and then visualized with 3,3′-diaminobenzidine (DAB). Negative controls were prepared using phosphate-buffered saline (PBS) instead of the respective primary antibody, while sections from tissues previously recognized as positive for the selected antibodies were used as positive controls.

All stained sections were observed and scored using a semiquantitative scale by two independent, blinded investigators (ZH Zhang & GX Song). The percentage of stained cells was recorded and each sample was classified according to a specific expression pattern for each antibody and the number of positive cells [28]. Staining of the cell membrane, cytoplasm or both in more than 10% of cells was considered as a sample that was positive for E-cadherin, N-cadherin and Vimentin, while >1% nuclear and/or cytoplasmic staining was considered positive for MDM2 and Snail [25].

### Animal xenograft model

This study was approved by the Animal Care and Use Committee of Nanjing Medical University. The nude mice were maintained in a controlled environment (temperature 20–25°C, humidity 50-80%, illumination 12 h light/12 h dark). Mice were fed standard laboratory food and water ad libitum for at least 1 week for environmental adjustment before study. 17β-Estradiol pellets (0.72 mg, 60-day release) were implanted subcutaneously into 5-week-old BALB/c female nude mice. One day after pellet implantation, MCF-7-MDM2 or MCF-7-pCMV cells were washed with serum-free medium and re-suspended in matrigel. The cell suspension (5×10^7^/ml, 0.2 ml) were injected subcutaneously into the right dorsal scapula region of the mice, n=6 mice/group. The general condition of the mice, including body weight and physical status, were observed daily. The mice were monitored by tumor growth. The tumor size was measured twice weekly for 6 weeks, and the tumor volumes (V) were calculated using the simplified formula length*width^2^*0.5. The mice were sacrificed by CO_2_ asphyxiation on day 42, and the tumors were resected, weighted and divided into three equal parts. One part of each tumor was formalin-fixed and paraffin-embedded for routine hematoxylin and eosin staining or immunohistochemical analysis. The other two parts of the tumor samples were subjected to western blotting and qRT-PCR analysis for MDM2, E-cadherin, N-cadherin, Vimentin and Snail expression. All animal studies were performed in accordance with the institutional guidelines.

### Statistical analysis

All data are representative of three experiments and are expressed as the mean ± SD. Relative gene expression data were analyzed using the 2^−ΔΔCT^ method [29]. Statistical significance for differences between groups was analyzed by one-way analysis of variance (ANOVA). Correlation analyses were performed using Spearman rank correlation analysis. All statistical tests were two-sided. Statistical significance was defined as P<0.05. All the statistical tests were performed using SPSS 13.0 statistical software.

## SUPPLEMENTARY FIGURES


