# Supplementary material for: Mouse double minute 2 (MDM2) upregulates Snail expression and induces epithelial-to-mesenchymal transition in breast cancer cells in vitro and in vivo
Source: Oncotarget. 2016 May 11;7(24):37177–91. doi: 10.18632/oncotarget.9287 (PMC5095067; doi:10.18632/oncotarget.9287)
Supplement: Supplementary file 1 [file oncotarget-07-37177-s001.pdf]

Mouse double minute 2 (MDM2) upregulates Snail expression and induces epithelial-to-mesenchymal transition in breast cancer cells *in vitro* and *in vivo*

SUPPLEMENTARY FIGURES

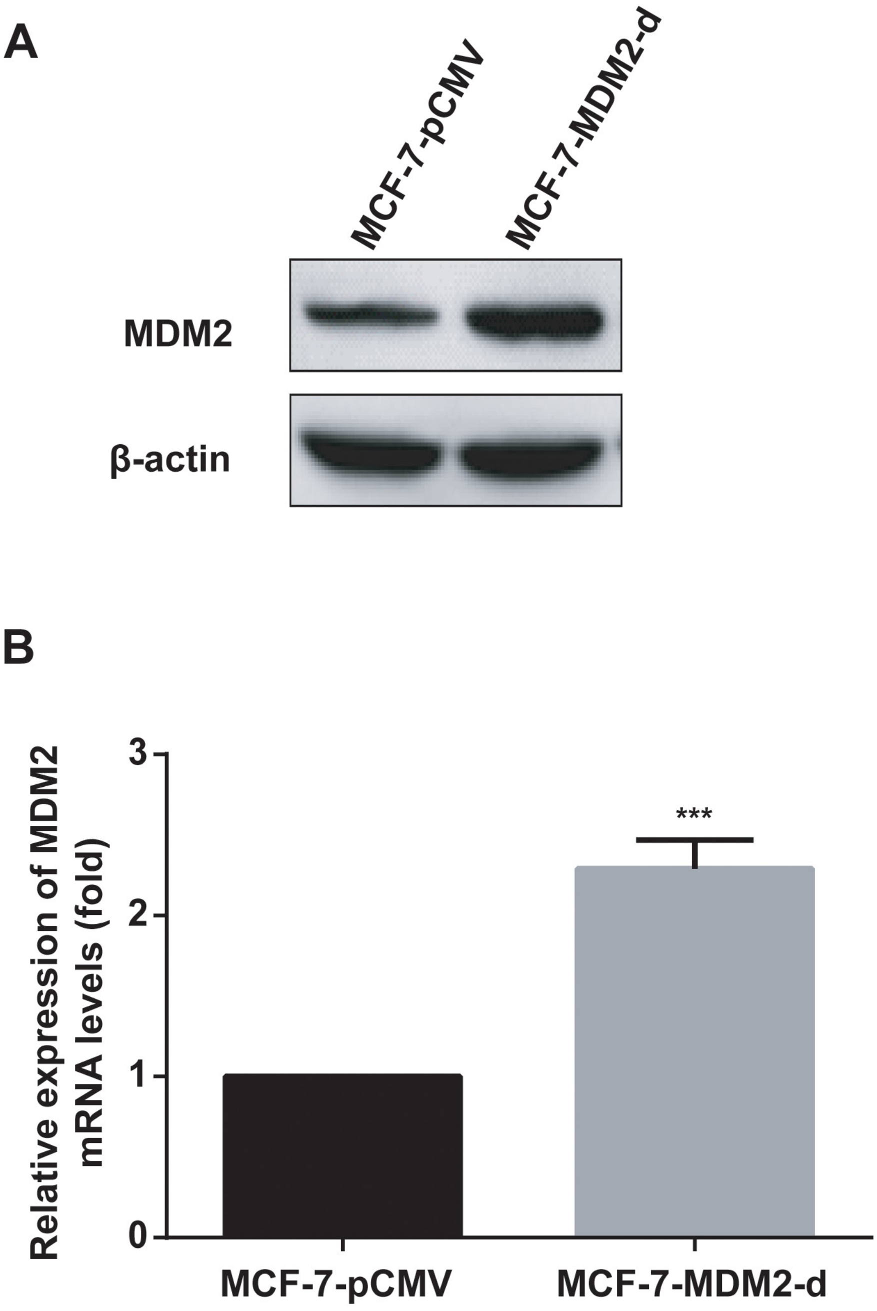

Supplementary Figure S1: Generation of stable cell lines. MDM2 protein expression was evaluated by western blotting in MCF-7-pCMV and MCF-7-MDM2-d cells **A.** β-actin was used as the loading control. *MDM2* mRNA expression was analyzed by qRT-PCR in MCF-7-pCMV and MCF-7-MDM2-d cells **B.** *GAPDH* served as an internal control. ***P<0.001. The results are from three independent experiments. Error bars indicate the standard deviation.

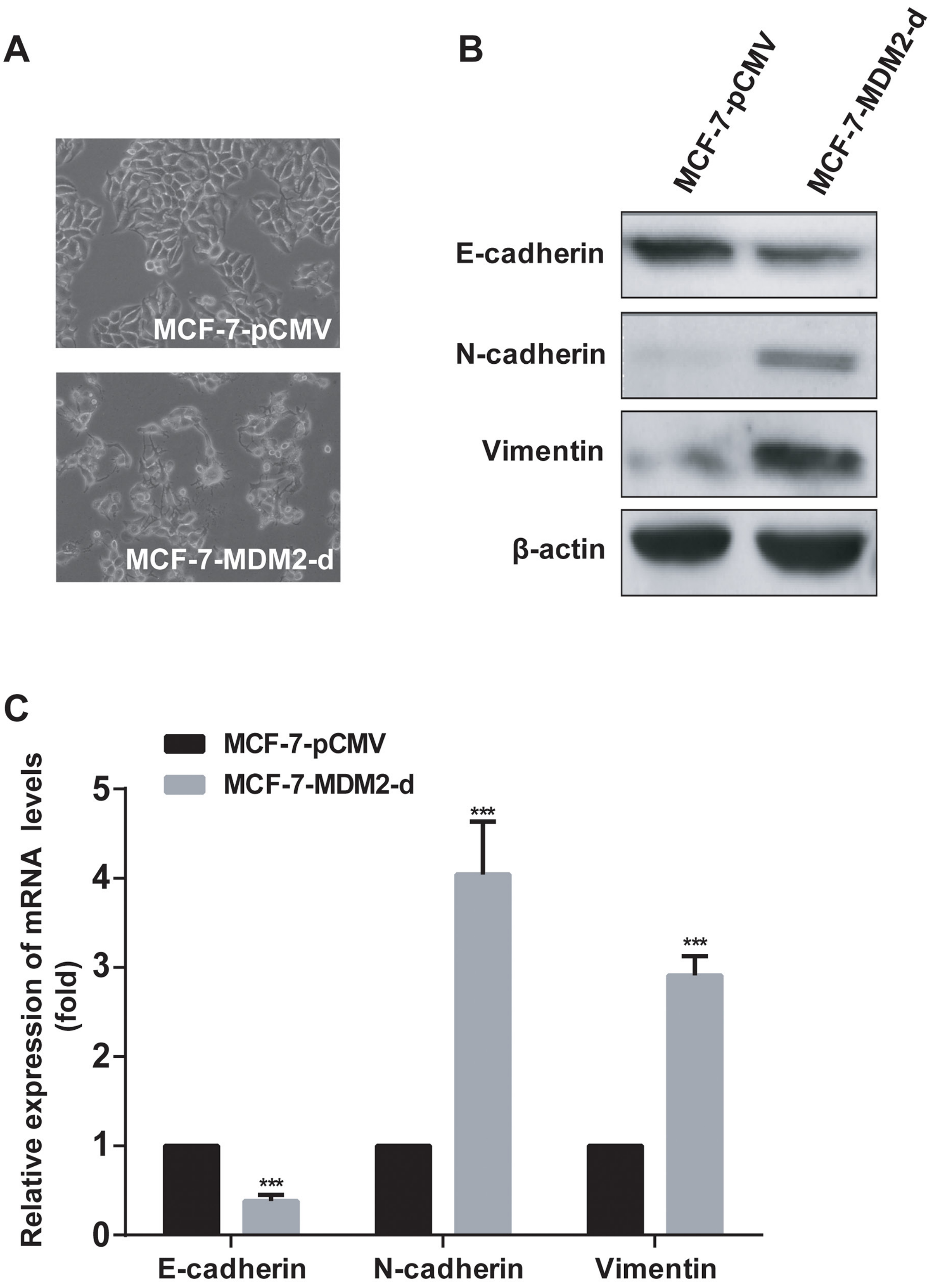

Supplementary Figure S2: MDM2 overexpression promotes EMT in MCF-7 cells. Representative phase-contrast images of MCF-7-pCMV and MCF-7-MDM2-d cells showed MDM2-related morphological changes **A.** (200×). Expression of epithelial and mesenchymal markers was evaluated by western blotting in MCF-7-pCMV and MCF-7-MDM2-d cells **B.** β-actin was used as the loading control. Expression of epithelial and mesenchymal markers was analyzed by qRT-PCR in MCF-7-pCMV and MCF-7-MDM2-d cells **C.** *GAPDH* served as an internal control. ***P<0.001. The results are from three independent experiments. Error bars indicate the standard deviation.

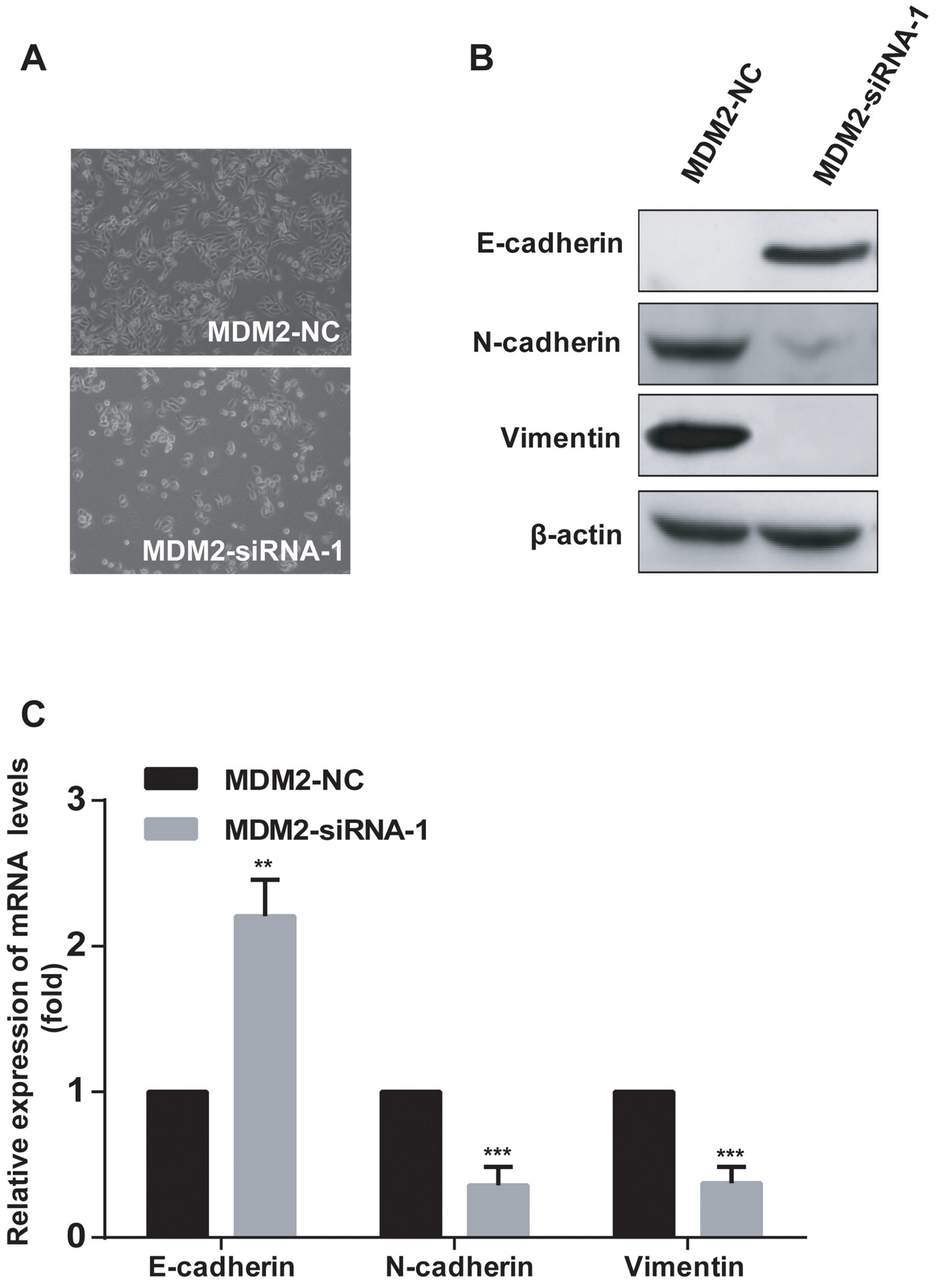

Supplementary Figure S3: *MDM2* knockdown promotes MET in MDA-MB-231 cells. Knockdown of *MDM2* in MDA-MB-231 cells induced morphological changes to an epithelial phenotype **A.** (200×). In 231-MDM2-siRNA-1 cells, qRT-PCR analyses showed increased E-cadherin and decreased N-cadherin and vimentin mRNA levels, compared with the control cells (231-MDM2-NC) **B.** In 231-MDM2-siRNA-1 cells, western blotting showed decreased N-cadherin and vimentin and increased E-cadherin protein levels **C.** $^{**}P<0.01$ and $^{***}P<0.001$. The results are from three independent experiments. Error bars indicate the standard deviation.
